# Pheromone communication among sexes of the garden cross spider *Araneus diadematus*

**DOI:** 10.1007/s00114-021-01747-9

**Published:** 2021-08-27

**Authors:** Andreas Fischer, Stefan Schulz, Manfred Ayasse, Gabriele Uhl

**Affiliations:** 1 Institute of Evolutionary Ecology and Conservation, Genomics University of Ulm, Albert-Einstein Allee 11, 89081 Ulm, Germany; 2 Department of Biological Sciences, Simon Fraser University, Burnaby, BC Canada; 3 Institute of Organic Chemistry, Technische Universität Braunschweig, Hagenring 30, 38106 Braunschweig, Germany; 4 General and Systematic Zoology, Zoological Institute and Museum, University of Greifswald, Loitzer Straße 26, 17487 Greifswald, Germany

**Keywords:** Sex pheromone, Electrophysiology, Female-female-interaction, Autodetection, Spider, Olfaction

## Abstract

Chemical communication plays a fundamental role in many aspects of an animal’s life from assessing habitat quality to finding mating partners. Behavioural observations show that chemical communication likewise plays an important role in spiders, but the contexts and the substances involved are little explored. Here, we investigate the chemical communication in the garden cross spider *Araneus diadematus* (Clerck, 1757) between and within the sexes. Using choice trials, we demonstrate that males are attracted to odours of adult females, but not to those of subadult females. Our data further suggest that adult females avoid odours of conspecific adult females, possibly in order to reduce reproductive competition with other females. Cuticle and silk extracts as well as headspace samples of subadult and adult virgin females were analysed via GC–MS. Available candidate compounds for the female sex pheromone were tested via electroantennography on palps (electropalpography) of adult virgin females and on females in behavioural trials. We propose sulcatone (6-methyl-5-hepten-2-one) as a candidate substance for the female volatile pheromone and several long-chained alkanes and alcohols as candidates for contact pheromones. Apart from demonstrating that attraction of males to females depends on the latter’s developmental stage, our study suggests that pheromones can also play an important role between females, an aspect that requires further attention.

**Supplementary Information:**

The online version contains supplementary material available at 10.1007/s00114-021-01747-9.

## Introduction

Communication consists of a sender emitting information via a signal and a receiver who reacts by changing its behaviour or physiology (Wilson 1975). The chemical communication channel, the oldest means of information transfer (Wyatt 2014), is used in a variety of contexts. Semiochemicals provide intra- and interspecific information that is related to mating, foraging, orientation or territories (e.g. Hölldobler et al. 2001; Mason and Parker 2010). In animals, semiochemicals attract prey (Eberhard 1977) or mates (Ayasse et al. 2001), provide kin recognition cues (Brennan and Kendrick 2006), may act as aggregation or warning signals (Savoie et al. 1998) and trigger worker reproduction in social insects (Rottler-Hoermann et al. 2016; Steitz and Ayasse 2020). In plants, semiochemicals are involved in pollinator attraction (Ayasse et al. 2011) and mediate interactions with herbivores and parasitoids (Turlings and Erb 2018).

Most studies on sex pheromones have been performed on insects (Symonds and Elgar 2008; Jacobson 2012) and there is ample information that females use pheromones to attract males (Ayasse et al. 2001; Ando et al. 2004; Harari and Steinitz 2013). Consequently, many sex pheromones have been identified in insects (Ayasse et al. 2001; Francke and Schulz 2010), e.g. in numerous species of butterflies, in which females attract males over long distances with only small amounts of sex pheromones (Ando et al. 2004). Males, in turn, have developed highly sensitive antennal receptors to receive the female sex pheromone (Roitberg and Isman 1992). Further, some female insects have been shown also to be capable of detecting female pheromones, suggesting that females can assess the presence of sexual rivals or even the density of signalling females in their habitat (Palanaswamy and Seabrook 1978; Harari et al. 2011).

For spiders, there is considerable behavioural evidence for the use of chemical communication for mate attraction and mate choice (Huber 2005; Gaskett 2007; Uhl and Elias 2011; Schulz 2013; Uhl 2013; Fischer et al. 2018b, 2020; Adams et al. 2021). Spider sex pheromones not only reveal the presence of a mating partner but may provide cues on age and feeding status of females, judging from differential male reactions (Cory and Schneider 2016, 2017, 2018, 2020; Jiang et al. 2018; Waner et al. 2018; Fischer 2019). One study reports on males producing a sex pheromone (Xiao et al. 2010), suggesting that mating systems in spiders are more diverse than assumed, and can entail reversed sex roles (Aisenberg et al. 2010). Whether pheromones have an effect on the behaviour of same sex individuals, however, has not been explored yet.

Electroantennography (EAG) or gas chromatography coupled with electroantennography (GC-EAD) has been used to investigate odour detection in a wide variety of insects (Roelofs 1984). In spiders, only two studies have been performed so far. Single sensillum recording showed a neural reaction in the spider *Cupiennius salei* when the sensilla were contacted with the sex pheromone (Tichy et al. 2001), and electro-antennography (EAG) on first leg tarsi (electrolegogram (Hebets and Chapman 2000)) of adult females demonstrated the existence of a male contact sex pheromone in *Pholcus beijingensis* (Xiao et al. 2010). So far, spider sex pheromones have been identified from only about 1% of all pheromone studies (Symonds and Elgar 2008). Spider pheromones were detected from the silk (Schulz and Toft 1993; Prouvost et al. 1999; Papke et al. 2000; Tichy et al. 2001; Xiao et al. 2009; Chinta et al. 2010; Jerhot et al. 2010; Fischer 2019), the cuticle (Prouvost et al. 1999; Chinta et al. 2010) or the headspace (Papke et al. 2001; Chinta et al. 2010) of live spiders.

As target species for investigating chemical communication between and within the sexes, we chose the orb weaver *Araneus diadematus* (Clerck 1757). The aims of our study were to test for the presence of a female-produced sex pheromone in *A. diadematus* and the reactions of males as well as females to the female odour. Furthermore, we attempted to identify candidate substances by comparing the chemical profiles of silk, cuticle and headspace samples from subadult (in their last moulting stage) and virgin adult females. Lastly, we established electroantennography by using pedipalps of female spiders as target structures—complementary to insect antenna.

## Methods

### Rearing of spiders

Subadult *Araneus diadematus* were collected near Ulm, Germany, in the early summer of 2014 and 2015. Spiders were kept individually in the laboratory in polypropylene plastic containers (17 × 17 × 8 cm) at 22 °C and in a natural light cycle. Subadult spiders were fed once a week with *Drosophila* sp., while adults were fed with house crickets *Acheta domesticus*. Females that matured in captivity were considered virgin and were used only after 7 days post maturity moult. A cotton ball in the lid of the container was moistened daily.

### Size measurements

We measured the tibia-patella length (in mm) of the first pair of legs as a parameter of body size. Measurements were performed with a HOREX® digital calliper (accuracy 0.01 mm) under a binocular microscope.

### Behavioural experiments

#### Dual-choice-tests

The Y-tube olfactometer (connecting arms: 9 cm length; single arm: 12 cm, inner diameter: 1.8 cm) experiment design is described in Fischer et al. (2018a, 2020). The setup was fixed in a polystyrene box (120 × 80 × 80 cm) and the only light source was a cold light lamp (Schott KL 1500 LCD, 2950 K) placed above the centre of the Y-tube. We alternated the stimuli sides throughout each experiment to compensate for any side bias. One-sample binomial tests were used to compare the observed choices against a theoretical probability. The null hypothesis for this test is that the results do not differ significantly from a frequency of 50:50. All statistical tests were performed with SPSS 26 statistical software and were performed two-tailed with alpha = 0.05.

For exploring choice behaviour of naïve males, first we tested if males exhibit a side preference in the Y-maze by offering a double blank control, i.e. two arms without test samples (Exp. 1, *N* = 20). In order to investigate if males can discriminate female developmental stages based on odour, we offered each male an adult virgin female versus a subadult female (one moult from maturity) (Exp. 2, *N* = 20). We also presented an adult virgin female versus an empty control (Exp. 3, *N* = 20). In each trial, we presented the same individuals as stimulus, with different individual stimuli across trials. We further tested with the olfactometer setup, if the cuticle extract of 10 adult virgin females contains the sex pheromone (Exp. 4, *N* = 20). For this, we offered 50 µl (0.5 adult female equivalent) of the cuticle extract versus 50 µl of the solvent methylene chloride (VWR, 99.8%) on filter paper. We allowed the solvent to evaporate for 3 min in the olfactometer before the spider was introduced.

In olfactometer experiments on females, we tested whether adult virgin females show differential responses to the odour of other females. We first tested whether females had a side preference by presenting double blank controls (Exp. 5, *N* = 20), then we offered adult females a choice between an adult virgin female versus a subadult female to investigate if females can discriminate between the two developmental stages based on volatile cues (Exp. 6, *N* = 20). In each trial, we presented the same individuals as stimulus with different individual stimuli across trials. We tested in another experiment, if the body size of a female (adult, virgin) influences her choice when offered a small and a large virgin female as a stimulus (Exp. 7, *N* = 20). Test females were categorised according to their body size (tibia-patella length) into two groups: smaller (*N* = 10, ${\overline{\mathrm{x}} }_{s}$ 5.30 ± 0.52 mm, min = 4.57 mm, max = 6.03 mm), and larger (*N* = 10, ${\overline{\mathrm{x}} }_{l}$ 6.78 ± 0.52 mm, min = 6.18 mm, max = 7.73 mm) than the mean size (6.04 ± 0.91 mm).

### Sample collection and chemical analysis

For the cuticle extracts, we randomly chose 22 adult virgin females and 22 subadult females and sacrificed them by freezing at − 20 °C overnight. Afterwards, they were placed in 500 µl methylene chloride (99.8%, VWR, Radnor, PA, USA), gently shaken for 3 min and removed. Half of the extract was concentrated to 50 µl using a gentle stream of nitrogen and later used for chemical analyses while the other half (250 µl) was used for bioassays. For the silk extracts, 17 adult virgin females and 14 subadult females were placed individually for 24 h in new plastic containers (17 × 17 × 8 cm) where they built the web. We reeled the silk with a clean metal stick and extracted the silk in 25 µl methylene chloride for 30 min at room temperature. The silk samples remained in the solvent for three weeks at − 18 °C.

For the headspace samples, we used self-made chromatoprobe filters that consisted of 1.5 mg Tenax-TA (mesh 60–80; Supelco, Bellefonte, PA, USA) and 1.5 mg Carbotrap B (mesh 20–40; Supelco, Bellefonte, PA, USA) (Dötterl and Jürgens 2005). Twenty-two adult virgin females and 21 subadults were individually placed in 200-ml glass vessels. With a membrane pump (DC12/16FK, FÜRGUT, Aichstetten, Germany), air was drawn for 8 h (flow 300 ml/min) through a charcoal pre-filter (50 mg, Supelco, Orbo 32 small) and the vessel containing the spider before passing through the chromatoprobe filter. We collected seven headspace controls by using empty vessels. All tools and containers were cleaned with ethanol (70%, Merck, Darmstadt, Germany), acetone (99.8%, Merck) and pentane (99%, Merck).

For the chemical analyses of the solvent extracts, 1 µl was injected splitless into an Agilent Technologies 7890A gas chromatograph with a DB-5 capillary column (30 m × 0.25 mm inner diameter, J&W) and a flame ionisation detector. We used hydrogen (2 ml/min) as the carrier gas. One minute after injection, the split valve was opened, and the temperature increased at a rate of 10°/min from 50 to 310 °C, at which it was held for 15 min. For quantitative assessment, 10 µl of *n*-heneicosane (stock solution 1 µg/1 ml) was added as an internal standard to each sample. Structural identification of candidate compounds was based on gas chromatography (Agilent Technologies 7890b) in combination with mass spectroscopy (Agilent Technologies 5977a MSD). The gas chromatographic condition was as above but with helium as the carrier gas (1.5 ml/min), instead of hydrogen. The MS interface was 150 °C and the ion source worked at 230 °C from m/z 35–450. We used the NIST library (2011 version) for structure elucidation.

For the headspace analyses, we used an Agilent Technologies 7820a gas chromatograph with a DB-5 capillary column (30 m × 0.25 mm inner diameter, J&W) and a flame ionisation detector. We used hydrogen (2 ml/min constant flow) as the carrier gas. One minute after injection, the split valve was opened and the temperature increased by 4°/min from 50 to 310 °C. The insertion was made with a thermodesorption unit (TDU, GERSTEL®). For quantitative analyses, 1 µl of *n*-heneicosane (stock solution 100 µg/1 ml) was added as an internal standard. Structure clarification of compounds was made similarly to extract samples using a GC–MS equipped with a thermodesorption unit.

### Statistical analysis of extracts, headspace samples and electrophysiological recordings

We inspected the GC profiles of 22 cuticle extracts of adult females, 22 cuticle extracts of subadult females, 17 silk samples of adult females, as well as 21 samples of the headspace of subadult and 22 samples of the headspace of adult females. We selected peaks that were consistently found in samples of adult females or peaks that were consistently higher in samples from adult than from subadult females. Because of too low concentrations of the female subadult silk compounds, silk extracts of subadults had to be excluded from chemical analysis. Peak areas were transformed into compositional data relative to the amount of all of the peaks of the run. Single principal component analyses (PCAs) were performed on the relative amounts of 55 selected peaks from the cuticle and silk extracts and 9 peaks from the headspace samples respectively. Based on the PCAs, 13 (extracts) and 5 (headspace) principal components (PCs) with an Eigenvalue greater than 1 were selected (Sramkova et al. 2008) and used to test for differences in odour bouquets of subadult and adult females using a discriminant function analysis (DFA). The standardised discriminant function coefficients greater than 0.7 and the factor loadings after varimax rotation were used to assess the importance of individual compounds. We considered a compound to have a high factor loading if the loading was above 0.5. We compared the relative amounts of compounds in extracts and headspace samples between subadults and adults using Mann–Whitney *U* tests and corrected *P*-values for multiple comparison using the Benjamini–Hochberg method (Chen et al. 2017) using the p.adjust function with method “fdr” in R (R Core Team 2020).

#### Air puff tests

Following chemical analysis, we tested whether females show a behavioural response towards compounds that we considered potential candidate substances for the sex pheromone (see chemical results). For this, we performed air puff experiments with synthetic compounds (Exp. 8, *N* = 11). Adult virgin females were kept in plastic boxes (17 × 17 × 8 cm) where they built webs over the course of 24 h. Each female was treated with a control air puff three times. Each puff was offered from a distance of 0.5 cm with an airflow of 1.5 l/min and lasted 0.5 s while the females were in the hub of their webs. The interval between puffs was ~ 3 s. After 30 min, each spider was treated again with puffs in the same manner; however, this time, the puffs contained the synthetic compound (treatment puff). The behaviour of the spider after every control and treatment puff was recorded by the observer. Each spider was tested with each one of the synthetic compounds in the same sequence. After exposure to a chemical stimulus, there is an interval of 1 to 5 days before a spider was exposed to the next synthetic compound, while control puffs always preceded the treatment puffs by 30 min. We used 5 µl of pure substances or 5 µl of a 10 µg substance/ml hexane solution, if the neat compound was solid. Chemicals were applied on 1-cm^2^ filter paper that was inserted into a glass Pasteur pipet through which the air was puffed. We recorded the following types of behavioural reactions: pedipalp drumming (drumming with pedipalps on the web), aggressiveness (both, lifting legs 1 + 2 and walking towards the puff source), huddling (adducting legs, similar to the position of dead spiders), retreating from the source (walking away from the stimulus), running (fast movement away from stimulus) and clinging to the web (contracting legs and pressing body on the web). Cases where spiders exhibited no reaction were also recorded. When females exhibited the same behaviour in response to both treatment and control puff, or if no reaction was observed, we recorded this as a “no reaction” response. If the response of the spider differed in type of behaviour between treatment and control, it was considered a reaction. The resulting binary data were analysed with a one-sample binominal test against an expected frequency of 50:50.

### Electrophysiological analyses

We adapted electroantennography for pedipalps of spiders, henceforth referred to as electropalpography (EPG), by attaching pedipalps to electrodes to detect bioactive compounds. We exclusively used female palps. Palps were detached from the body at the tibia-patella joint and placed between two glass capillaries filled with spider ringer solution (160 mM NaCl, 7.5 mM KCl, 1 mM MgCl_2_, 4 mM NaHCO_3_, 4 mM CaCl_2_, 20 mM glucose, pH 7.4) (Groome et al. 1991). The capillaries were placed between two electrodes. The amplitude of the EPG was measured with an IDAC-2 amplifier and recorded with the software EAGpro (V2, 2015, Syntech, Kirchzarten, Germany). The chemical stimuli were puffed into the air flow through a Pasteur pipette containing 4 µl of the pure compound. For 1-dodecanol, we used 4 µl of a 10 µg/ml hexane solution. In those cases, in which the synthetic compounds were dissolved in solvent, we performed solvent control trials on the same pedipalp, followed by a test to control if the vitality of the pedipalp and consequently the sensitivity of the receptors decreased during the test by comparing control puffs before and after a treatment. Amplitude heights resulting from the chemical stimuli were compared statistically against the reactions towards the control air puffs using Wilcoxon matched pair tests. The amplitude heights were measured with the software EAGpro 2.0 (Syntech 2015).

## Results

### Attractiveness of adult versus subadult females for males

**Fig. 1:**
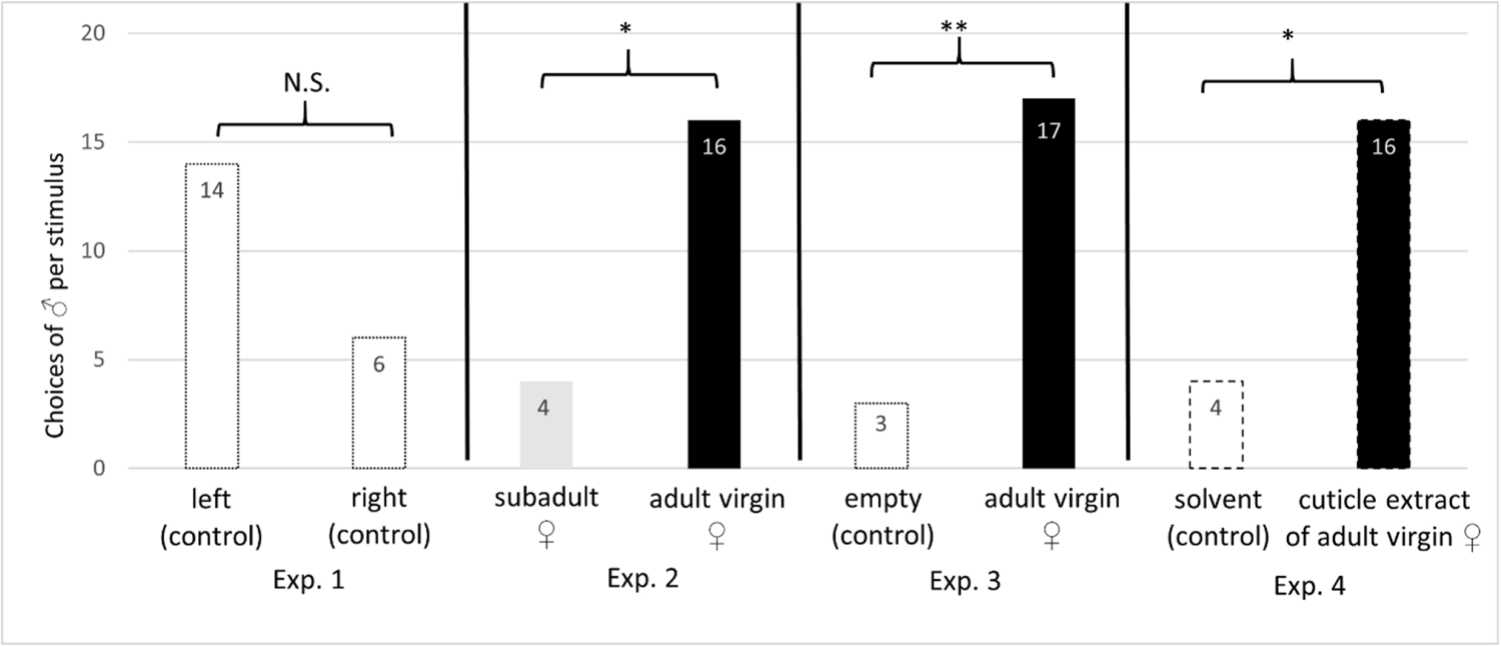
Attractiveness of various odour samples of subadult and adult females of *Araneus diadematus* for *males* in a Y-tube olfactometer tests. Exp. 1: side preference test (double blank treatment, *N* = 20); Exp. 2: test of discrimination of female developmental stage (odour of a subadult female versus that of an adult female, *N* = 20); Exp. 3 test for existence of a volatile sex pheromone (odour of an adult female versus blank control, *N* = 20); Exp. 4: test for presence of the volatile sex pheromone in the cuticle extract (cuticle extract of adult females versus solvent control, *N* = 20); * *p* < 0.05; ** *p* < 0.01; N.S. = not significant, binomial tests

In the olfactometer tests, males did not show a significant side preference in the double blank controls (14 vs. 6, binomial test, *N* = 20, *p* = 0.115, Fig. 1, Exp. 1), and significantly preferred the odour of live virgin adult to the odour of subadult females (16 vs*.* 4; binomial test, *N* = 20, *p* = 0.012, Fig. 1, Exp. 2). Furthermore, males significantly preferred the odour of adult virgin females to an empty control (17 vs. 3; binomial test, *N* = 20, *p* = 0.003, Fig. 1, Exp. 3). Additionally, when presenting cuticle extract of adult virgin females versus the solvent control, males preferred the cuticle extract (16 vs. 4; binomial test, *N* = 20, *p* = 0.012, Fig. 1, Exp. 4).

### Female-female interaction

**Fig. 2:**
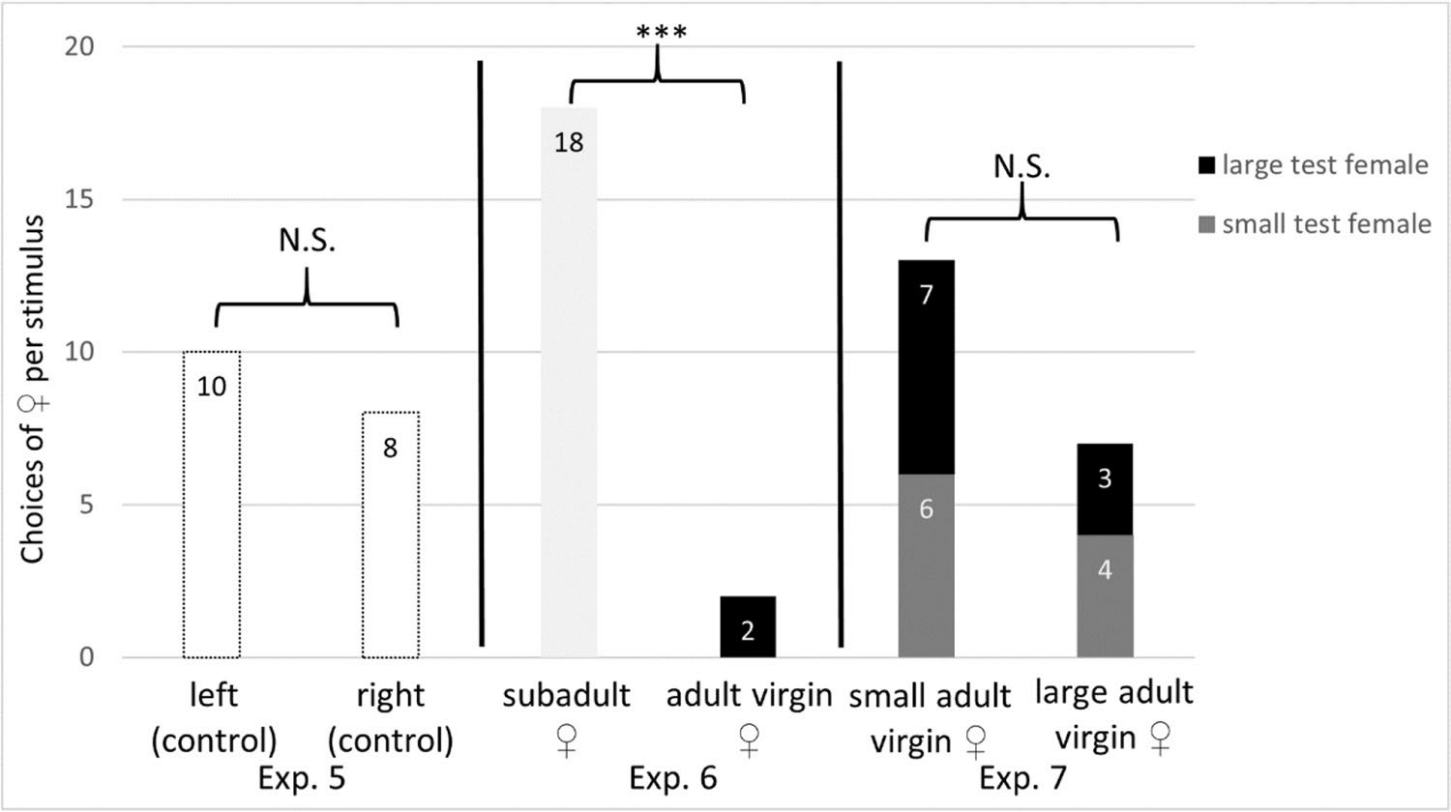
Attractiveness of various odour samples of subadult and adult females of *Araneus diadematus* for *females* in Y-tube olfactometer tests. Exp. 5: side preference test (double blank treatment, *N* = 20); Exp. 6: test of discrimination of female developmental stage (odour of a subadult female versus that of an adult female, *N* = 20); Exp. 7: test of discrimination of female size based on odour (odour of a small adult female versus that of a large adult female, *N* = 20) ***p < 0.001; N.S. = not significant, binomial test (Exps. 5 + 6) or fisher exact test (Exp. 7)

We explored whether female *A. diadematus* react differentially to conspecific females of different developmental stages (adult virgin versus subadult females) in an olfactometer test. As in the trials with males, females did not show a side preference in the empty setup (10 vs. 8; binomial test, *N* = 20, *p* = 1.000, Exp. 5, Fig. 2). Females significantly preferred conspecific subadult females to adult virgin ones (18 vs. 2; binomial test, *N* = 20, *p* < 0.001, Exp. 6, Fig. 2). Females did not distinguish between small and large adult virgins (13 vs. 7; binomial test, *N* = 20, *p* = 0.263, Exp. 7, Fig. 2). The size of the test animal did not significantly affect its decision; as of the 10 large females, seven preferred the small female while three preferred the large female. Of the 10 small females, six preferred the small female stimulus while four approached the large female (Fisher exact test, *N* = 20, *p* = 1.000, Exp. 7, Fig. 2).

### Chemical analyses

**Fig. 3:**
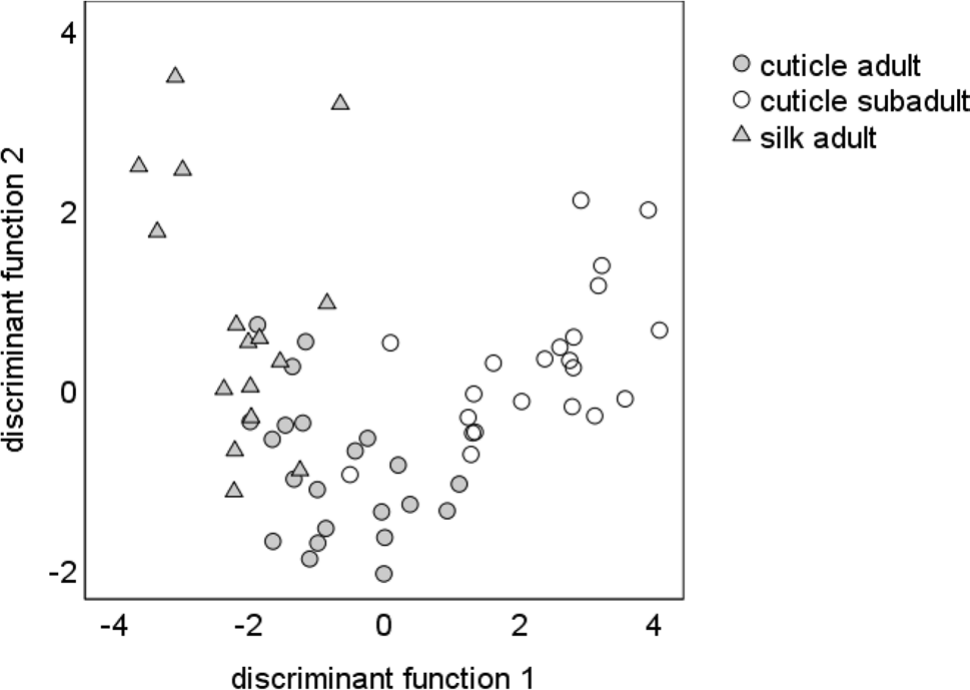
Comparison of cuticle extracts of adult and subadult females and silk from adult females of *Araneus diadematus* with a canonical discriminant function analysis. We found a significant difference between groups. *N* = 22 each; discriminant function (df) 1: *χ*^2^ = 89.75, df = 26, *p* < 0.001 explained 86.4% of variance, while df 2 *χ*^2^ = 22.28, df = 12, *p* = 0.035 explained 13.6% of variance. 83.3% of the females could be correctly classified according to their odour bouquets

**Fig. 4:**
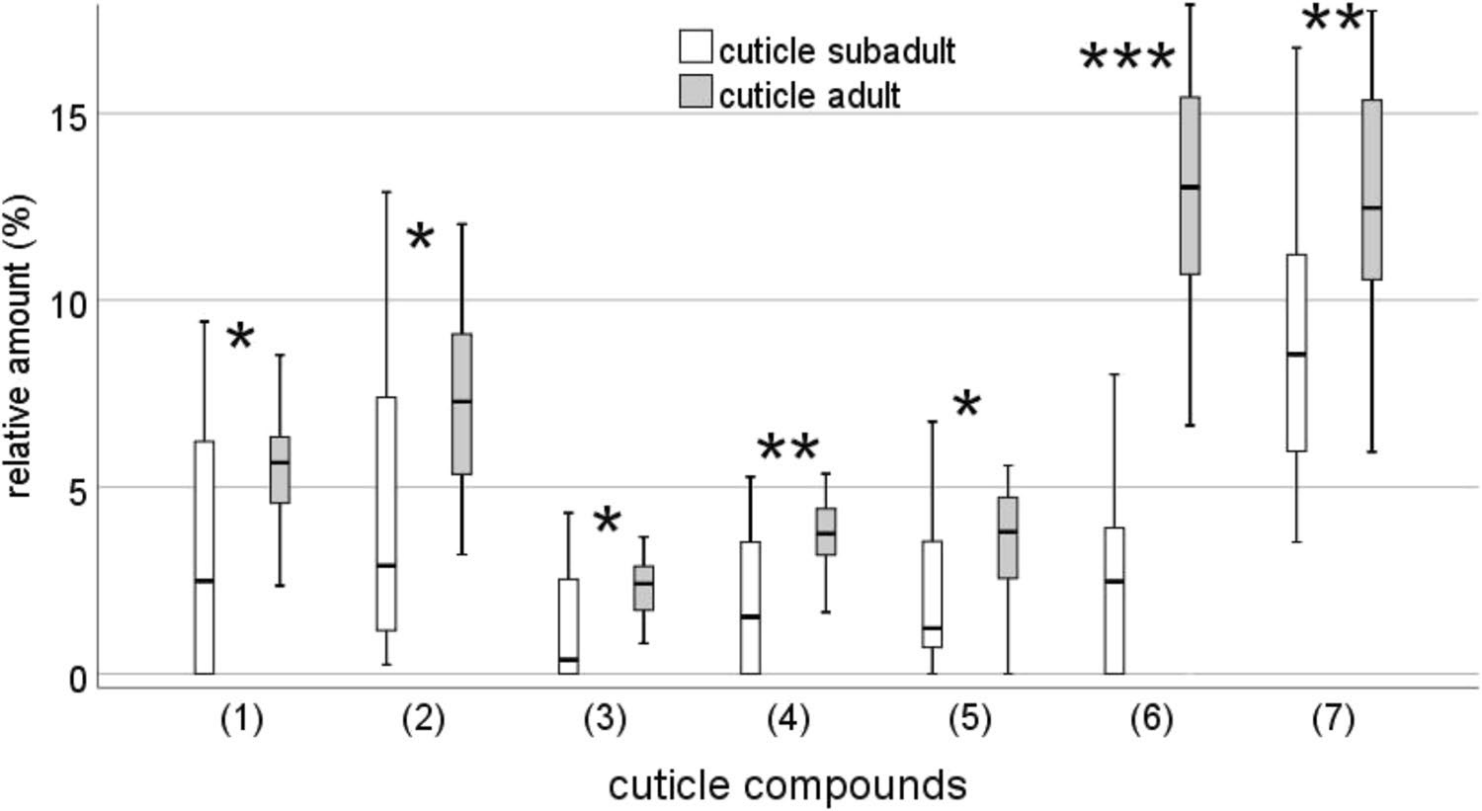
Relative amounts (in %) of selected alcohols and alkanes in cuticle extracts of subadult and adult females of *Araneus diadematus* (*N* = 22 each). Selection was based on the degree to which substances contribute to group separation in a DFA. (1) Unknown alcohol; (2) dimethyloctacosane and 3-methyloctacosane; (3) unknown alcohol; (4) 5,15 and 5,17 dimethylhentriacontane; (5) triacontane; (6) hentriacontane; (7) tritriacontane; *p < 0.05; **p < 0.01; ***p < 0.001, Mann–Whitney *U* tests

**Fig. 5:**
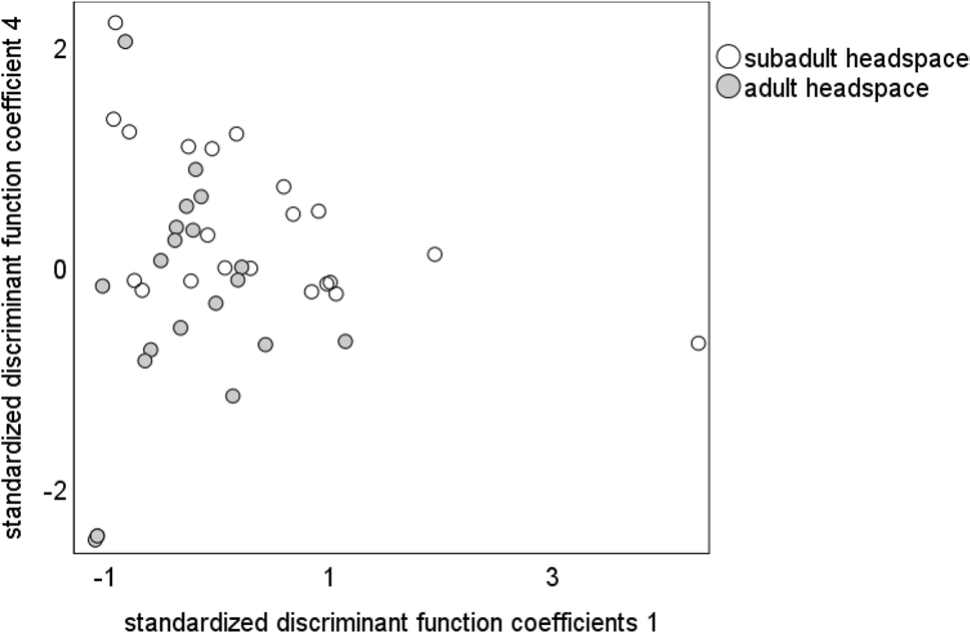
Comparison of headspace samples in subadult and adult females of *Araneus diadematus* with a canonical discriminant function analysis. *N*_subadult_ = 21, *N*_adult_ = 22, single discriminant function 1: *χ*^2^ = 28.16, df = 5, *p* < 0.001. 90.7% of the females could be correctly classified according to their odour patterns

A single principal component analysis (PCA) of adult and subadult cuticle and adult silk extracts based on the relative amounts of 55 compounds (suppl. Table 1) resulted in 13 PCs with an Eigenvalue above one, explaining 86.76% of the total variance. The canonical discriminant function analysis (DFA) significantly separated the samples of adult virgin females from those of subadults with respect to the relative amount of chemical compounds in their cuticle extracts. Discriminant function 1 (*χ*^2^ = 89.75, df = 26, *p* < 0.001) explained 86.4% of variance, while discriminant function 2 (*χ*^2^ = 22.28, df = 12, *p* = 0.035), explained 13.6% of variance (Fig. 3). 83.3% of the females could be correctly classified according to their odour bouquets. Discriminant function 1 separated subadult from adult females, while discriminant function 2 separated cuticle and silk extracts of adult females. The standardised discriminant function coefficients and the component loadings in a PCA revealed that seven alkanes and two alcohols are most important for discrimination of female groups and silk (suppl. table 1). On the cuticle of adult females, dimethyloctacosane and 3-methyloctacosane (Retention Index (RI) = 2883, Mann–Whitney *U* test, *U* = 136, *N*_1_ = *N*_2_ = 22, *p* = 0.013, Fig. 4), triacontane (RI = 2998 *U* = 133.5, N_1_ = *N*_2_ = 22, *p* = 0.013, Fig. 4), hentriacontane (RI = 3096, *U* = 42, *N*_1_ = *N*_2_ = 22, *p* < 0.001, Fig. 4) and 5,15 and 5,17 dimethylhentriacontane (RI = 3177, *U* = 98, *N*_1_ = *N*_2_ = 22, *p* = 0.002, Fig. 4), tritriacontane (RI = 3297, *U* = 99, *N*_1_ = *N*_2_ = 22, *p* = 0.002, Fig. 4) as well as two unknown alcohols (RI = 3116, *U* = 128, *N*_1_ = *N*_2_ = 22, *p* = 0.011 and RI = 3316, *U* = 129, *N*_1_ = *N*_2_ = 22, *p* = 0.011, respectively; Fig. 4) were more abundant compared to the cuticle of subadult females.

**Fig. 6:**
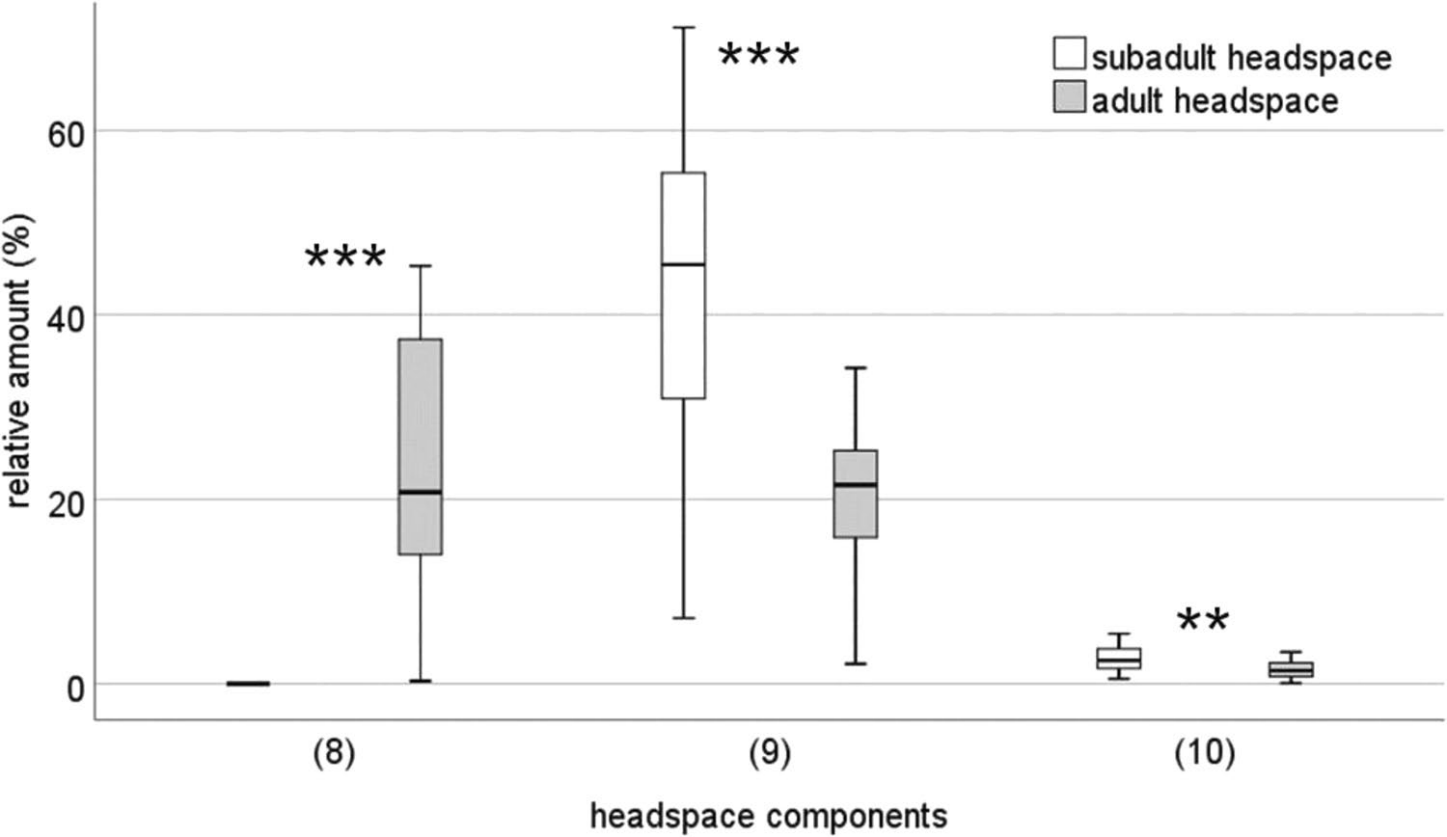
Relative amounts of selected compounds in headspace samples of subadult (*N* = 21) and adult females (*N* = 22) of *Araneus diadematus*. Selection was based on the degree to which substances contribute to group separation in a DFA. (8) sulcatone; (9) 1-dodecanol; (10) tricosane; **p < 0.01; ***p < 0.001, Mann–Whitney *U* tests

For the headspace samples, a PCA based on the relative amounts of nine compounds (suppl. table 2) resulted in five PCs with an eigenvalue above one, explaining 86% of the total variance of the headspace profile. The canonical DFA significantly separated both female groups (adult and subadult) with respect to the relative amounts of chemical compounds in their headspace (discriminant function 1: *χ*^2^ = 28.16, df = 5, *p* < 0.001, Fig. 5). 90.7% of the females could be correctly classified according to their odour patterns. According to standardised discriminant function coefficients and the components loadings, a ketone and an alcohol and an alkane are responsible for the statistical separation of subadult and adult females. Twenty-two adult and 21 subadult female samples were tested for differences in their relative amounts of compounds (relative within a sample). Sulcatone occurred only in adult and not in subadult females (RI = 989, Mann–Whitney *U* test, *U* = 0, *N*_1_ = 22, *N*_2_ = 21, *p* < 0.001, Fig. 6) and was considered a strong candidate compound for the sex pheromone. On the other hand, 1-dodecanol and tricosane were present in lower amounts in adult than in subadult females (RI = 1473, Mann–Whitney *U* test, *U* = 385, *N*_1_ = 22, *N*_2_ = 21 *p* < 0.001 and RI = 2296, *U* = 339, *N*_1_ = 22, *N*_2_ = 21 *p* = 0.009, respectively, Fig. 6). Since 1-dodecanol declined strongly from subadult to adult females, we focussed on this compound for further testing.

### Tests with compound puffs and controls

Spiders exhibited significant behavioural responses to treatment puffs with sulcatone (6-methyl-5-hepten-2-one) when compared to control air puffs. Sixteen females altered their behaviour when puffed with sulcatone compared to the control puff whereas four females did not show a change in behaviour (binomial test, *N* = 20, *p* = 0.012). Specifically, five females drummed with their pedipalps, two lifted leg pairs 1 + 2 and walked towards the treatment, four huddled, three retreated from the odour source, one ran to the periphery of the web and one clung to the web. In contrast, when 1-dodecanol was offered, two of 11 females changed their behaviour compared to the control puff (binomial test, *N* = 11, *p* = 0.030). Those that showed a reaction (*n* = 2) drummed with the pedipalps or clung to the web. Since 1-dodecanol was dissolved in hexane, we also tested hexane against air. Hexane did not elicit a significant response in females, as only five of 11 females reacted to hexane: two with pedipalp drumming, two with aggressive behaviour and one clung to the web (binomial test, *N* = 11, *p* = 0.161).

### Electrophysiological analyses

The electrophysiological responses of female pedipalps revealed a significantly stronger receptor response, measured as amplitude height, for pure sulcatone and the 1-dodecanol hexane solution compared with the control (air) (Table 1, suppl. Figure 1). As dodecanol was diluted in hexane, we compared amplitude heights of dodecanol and hexane, which were significantly different (Table 1). We found no significant difference in amplitude between the puffs before and after a treatment (Table 1) suggesting that receptors did not change their vitality. Since only 5% of palps responded in our setup, the overall sample size for the electrophysiological tests was low.

**Table 1:** Effects of sulcatone, 1-dodecanol, a solvent control (hexane) and an air control on amplitude height in electropalpography. *N* = the number of puffs of a stimulus on a palp. Prior to puffs containing a specific compound, control air puffs were performed. Electro-palpal responses were recorded for both compounds and air control puffs. Air-control puffs compared puffs before and after testing the solvent control to test for habituation. Statistical significance of responses were tested using Wilcoxon matched pairs tests

	***N***	**Amplitude height** $\overline{\mathbf{x} }$_**air**_** ± SD [mV]**	**Amplitude height** $\overline{\mathbf{x} }$_**scompound**_** ± SD [mV]**	***Z*, *p***
**Sulcatone**	16	2.35 ± 1.20	− 0.30 ± 0.10	− 3.52, < 0.001
**1-Dodecanol + hexane**	30	− 0.06 ± 0.01	− 0.04 ± 0.01	3.75, < 0.001
		$\overline{\mathbf{x} }$_**hexane**_ ± SD	$\overline{\mathbf{x} }$_**1-dodecanol**_ ± SD	
**Hexane control**	21	− 0.52 ± 0.22	− 0.04 ± 0.013	4.02, < 0.001
		$\overline{\mathbf{x} }$_**air1**_ ± SD	$\overline{\mathbf{x} }$_**air2**_ ± SD	
**Habituation-control**	28	− 0.06 ± 0.01	− 0.05 ± 0.02	1.21, 0.225

## Discussion

Exploring the chemical communication of *A. diadematus* revealed that odours from adult females attract males, while adult females seem to avoid adult female odour. The electrophysiological recordings and behavioural tests in which we used compounds identified from headspace analysis suggest that sulcatone seems to be a key candidate for a volatile sex pheromone compound. Sulcatone might be complemented by long-chained alkanes that might play a role during contact chemoreception in this species. We tested pheromone candidates on females, demonstrating that pheromones are perceived by same sex individuals.

Male *A. diadematus* preferred adult virgin females and their cuticle extract over the respective controls (Exps. 3 + 4, Fig. 1) and subadult females (Exp. 2, Fig. 1), indicating the presence of a volatile pheromone on the cuticle surface of adult virgin females. Subadult females likely do not produce a sex pheromone, as was found in other studies on spiders (Fischer 2019). Such chemical crypsis might be beneficial since signalling can cause costs, not only through the production of the compound but via kleptoparasitism or reduced prey-capture rates due to the presence of males in and around the web (Erez et al. 2005; Fischer et al. 2018b).

Olfactory signals may not only be used in the inter-sexual communication but also in a female-female context. Female *A. diadematus* seem to avoid adult virgin females (Exp. 6, Fig. 2). Air puff exposure (Exp. 8) and electrophysiological recordings (Exp. 9) indicate that adult females indeed perceive the candidate sex pheromone of other females. The size of the adult female stimulus did not affect the choice of the test female, nor did her own size (Exp. 7, Fig. 2). Female *A. diadematus* readily attack and kill web-invading (conspecific) females (A.F. pers. observation), which might explain the observed avoidance behaviour towards adult females. Alternatively, intrasexual competition for mates may also explain our findings, if fitness benefits decrease with increasing numbers of signalling females. Thus far, there is little information on the prevalence of intrasexual female competition based on pheromones. The response of the pheromone producing sex (usually female) to the sex pheromone has been termed autodetection (Holdcraft et al. 2016). We find this term misleading since it might suggest self-detection instead of detecting same sex individuals. In some butterflies, it has been shown that the female sex pheromones induce dispersal of females (e.g. Palanaswamy and Seabrook 1978; Sanders 1987) or lead to changes in the calling behaviour (e.g. Palanaswamy and Seabrook 1985; Weissling and Knight 1996; Gökçe et al. 2007). In the lepidopteran *Grapholita molesta*, exposure to the conspecific female pheromone resulted in reduced female mating success (Kuhns et al. 2012). Intrasexual competition of females mediated by olfactory information should be investigated in future studies. Investigating the calling investment of females exposed to the odour of conspecific females of different quality will further enhance our understanding the communication dynamics between females.

Chemical substances on the cuticle and in the headspace of adult virgin females differed significantly from those of subadult females. The silk and cuticle of adult spiders were similar in their chemical profile (Fig. 3; discriminant function 1) suggesting that the sex pheromones and/or cues that indicate maturity might be present on both the cuticle and the silk though further investigations are necessary to support this assumption. Silk of subadult females had to be excluded from our analysis as compounds were under the detection-limit of our analytical equipment. As to the cuticle extracts, long-chained methyl- and *n*-alkanes such as dimethyloctacosane and 3-methyloctacosane, triacontane, hentriacontane, 5,15 and 5,17 dimethylhentriacontane and tritriacontane as well as two unknown alcohols were statistically responsible for the separation of subadult and adult females (Fig. 3). Methylated alkanes as sex pheromones in spiders have not been reported yet (Schulz 2013). Long-chained methylated alkanes may function as both contact and volatile pheromones (Mukherjee et al. 2013). Here, we propose these alkanes as candidate sex pheromone components of *Araneus diadematus*. Bioassays with synthetic compounds in naturally occurring amounts need to be performed in order to test the biological relevance of these compounds.

The headspace analysis also demonstrates that subadult and adult females differed in their chemical profiles. Only adult females contained sulcatone, suggesting that sulcatone is not produced before females reach their final moult. Electrophysiological investigations and the air puff bioassay suggest sulcatone as a candidate for the sex pheromone. The overall level of the alcohol 1-dodecanol is reduced in adults compared to subadults and in 82% of the puff tests the spiders did not react. Therefore, it is unlikely that 1-dodecanol acts as a sex pheromone although it was electrophysiologically active and thus should be tested in future studies. Likewise, the alkane tricosane was found more abundant in subadults than in adult females rendering it an unlikely candidate for a sex pheromone. All electrophysiological analyses were performed with palps from female *A. diadematus*. Males in this species are shorter lived and were not available at the time of testing. We hypothesise that males react to the tested substances but further analysis might reveal differences between the sexes.

Interestingly, we found little overlap between the identified compounds from the extracts and headspace analyses (suppl. tables 1 + 2) indicating that the two methods collect different chemical information. Mostly longer chained methylated alkanes were found on the cuticle and silk extracts, while more volatile compounds were identified in the headspace samples. So far, nothing is known about the pheromone production site in spiders. It has been suggested that silk glands might be involved (Schulz 2004) as the pheromones can be found on the silk that has been drawn straight from the silk glands (Tichy et al. 2001). If the silk glands indeed are the source of pheromone production, it remains to be investigated how the pheromone is transferred to the cuticle.

Our study shows that silk extracts alone would suffice for future pheromone studies, as both cuticle and silk of adult spiders were almost identical when compared with material derived from subadults (discriminant function 1). No observed compound was unique to either cuticle or silk extracts. Silk can be studied over time from the female’s final moult to late in the reproductive season, thus becoming available as a tool to detect the dynamics of pheromone production and its modulation through mating events or socio-sexual contexts. Using headspace sampling and silk extracts should allow the identification of both contact and volatile pheromone components, as was done in Chinta et al. 2010.

We applied electrophysiology for the identification of a semiochemical candidate in combination with bioassays. However, since an electrophysiological response was difficult to obtain, future investigations require optimising the electro palpogram (EPG) setup. In contrast to insect chemosensory sensilla, electrophysiology on spider sensilla is hampered because of their multimodal character. In spiders, the known chemosensory sensilla are tip-pore sensilla that possess chemoreceptive cells whose dendrites are found in the shaft as well as several mechanoreceptive cells at their base (Tichy et al. 2001; Müller et al. 2020). EPG or GC-EPD are recording the sum potential of several sensilla under a constant airflow, stimulating also the mechano-receptors in the sensilla. Since Tichy and coauthors (2001) were able to differentiate signals from the mechanoreceptor and the contact-chemoreceptor of a single sensillum, separation of the modalities is possible in principle. Future single sensillum recordings (SSR) in combination with GC-EPD (or electrolegograms) and GC–MS will clarify which of the chemosensory sensilla of spiders perform gustation and/or olfaction and which compounds are involved. A sensillum map of the orb-weaver *Argiope bruennichi* (Ganske and Uhl 2018) showed that only one type of candidate sensilla (tip-pore sensilla) can be found on pedipalps and legs of both sexes in this species. This map can be used as a basis for exploring whether these sensilla can perform gustation as well as olfaction or whether there are regional functional differences between seemingly similar sensilla.

## Conclusion

We suggest that male *A. diadematus* approach adult females based on sulcatone as the major candidate compound of the volatile sex pheromone. Due to their low volatility, several long-chained methyl and *n*-alkanes along with two unknown alcohols might be candidates for contact sex pheromones of *A. diadematus*. Our data further suggest that female spiders react to female odours, which opens new avenues for the study of intrasexual competition based on conspecific odour and advances our insight into the diverse facets of chemical communication.

## Supplementary Information

Below is the link to the electronic supplementary material.

Supplementary file1 (DOCX 142 KB)

## Data Availability

The data will be uploaded to the Dryad data repository.
