# Supplementary material for: Pheromone communication among sexes of the garden cross spider Araneus diadematus
Source: Naturwissenschaften. 2021 Aug 27;108(5):38. doi: 10.1007/s00114-021-01747-9 (PMC8397638; doi:10.1007/s00114-021-01747-9)
Supplement: Supplementary file 1 — Supplementary file1 (DOCX 142 KB) [file 114_2021_1747_MOESM1_ESM.docx]

**Pheromone communication among sexes of the garden cross spider *Araneus diadematus***

Andreas Fischer^*^, Stefan Schulz, Manfred Ayasse and Gabriele Uhl

**Supplementary table 1:**

Cuticle and silk compounds of adult and subadult *Araneus diadematus* females. These compounds were included in a principial component analysis, followed by a discriminant function analysis. Compounds that are characteristic to a developmental stage were considered sex pheromone candidates.

X in name denotes unknown position.

| **#** | **Compound** | **Retention Index** |
| --- | --- | --- |
| 1 | benzyl alcohol | 1041 |
| 2 | octadecanal | 2023 |
| 3 | butyl stearate | 2186 |
| 4 | docosane | 2200 |
| 5 | tricosane | 2298 |
| 6 | 9-methyltricosane | 2334 |
| 7 | butyl octadecenoate | 2383 |
| 8 | tetracosane | 2396 |
| 9 | 2-methyltetracosane | 2463 |
| 10 | pentacosane | 2496 |
| 11 | hexacosane | 2596 |
| 12 | 9-, 10-, 11-, 12-, 13-methylhexacosane, mixture | 2631 |
| 13 | 2-methylhexacosane | 2659 |
| 14 | 3-methylhexacosane | 2669 |
| 15 | heptacosane | 2696 |
| 16 | 11- + 13-methylheptacosane | 2729 |
| 17 | 2-methylheptacosane | 2759 |
| 18 | 3-methylheptacosane | 2770 |
| 19 | octacosane | 2797 |
| 20 | 9-, 10-, 11-, 12-, 13-, 14-methyloctacosane | 2829 |
| 21 | 2-methyloctacosane | 2863 |
| 22 | dimethyloctacosane and 3-methyloctacosane mixture | 2883 |
| 23 | nonacosane | 2897 |
| 24 | 11-,13-,15 methylnonacosane and 9-methylnonacosane, mixture | 2932 |
| 25 | 5-methylnonacosane | 2947 |
| 26 | 2-methylnonacosane and 9,13 dimethylheptacosane, mixture | 2960 |
| 27 | 3-methylnonacosane | 2971 |
| 28 | triacontane | 2998 |
| 29 | 3,13-dimethylnonacosane and 3,X-dimethylnonacosane, mixture | 3009 |
| 30 | 10-, 11-, 12-, 13-, 14-, 15-methyltriacontane | 3028 |
| 31 | 2-methyltriacontane | 3059 |
| 32 | 2,X dimethyltriacontane | 3092 |
| 33 | hentriacontane | 3096 |
| 34 | unknown alcohol | 3116 |
| 35 | 9-, 11-, 13-, 15-methylhentriacontane | 3131 |
| 36 | 13-,17-dimethylhentriacontane and 11-, 15-dimethylhentiacontane and 9-,13-dimethylhentriacontane, mixture | 3156 |
| 37 | 5,15-, 5,17-dimethylhentriacontane | 3177 |
| 38 | 3,15-, 3,17-dimethylhentriacontane | 3202 |
| 39 | 10-, 11-, 12-, 13-, 14-, 15-, 16-methyldotriacontane | 3228 |
| 40 | 13-,17-dimethyldotriacontane and 11,X-dimethyldotriacontane mixture | 3249 |
| 41 | hexadecyl hexadecanoate, branched | 3289 |
| 42 | tritriacontane | 3297 |
| 43 | unknown alcohol | 3316 |
| 44 | 11-, 13-, 15-, 17-methyltritriacontane | 3334 |
| 45 | 13-,17-dimethyltririacontane and 11-, 15-dimethyltritriacontane and 9-, 13-dimethyltritriacontane mixture | 3353 |
| 46 | 5,15-, 5,17-dimethyltritriacontane | 3377 |
| 47 | 3,15-, 3,17-dimethyltritriacontane | 3401 |
| 48 | 11-, 12-, 13-, 14-, 15-, 16-, 17-methyltetratriacontane | 3428 |
| 49 | 13,17-dimethyltetratriacontane and 15,19-dimethyltetratriacontane | 3452 |
| 50 | 11-, 13-, 15-, 17-methylpentatriacontane | beyond C-standard |
| 51 | 13,17-, 15,19-dimethylpentatriacontane | beyond C-standard |
| 52 | unknown hydrocarbon | beyond C-standard |
| 53 | unknown hydrocarbon | beyond C-standard |
| 54 | 13-, 14-, 15-, 16-, 17-, 18-methylhexatriacontane | beyond C-standard |
| 55 | X,X-dimethylhexatriacontane | beyond C-standard |

**Supplementary table 2:**

Volatile compounds of the headspace of adult and subadult *Araneus diadematus* females. The relative amounts of these compounds were included in a principial component analysis followed by a discriminant function analysis. Compounds that are characteristic to a developmental stage were considered sex pheromone candidates.

| **#** | **Compound** | **Retention Index** |
| --- | --- | --- |
| 1 | sulcatone | 989 |
| 2 | tridecane | 1299 |
| 3 | 1-dodecanole | 1473 |
| 4 | unknown compound | 1919 |
| 5 | methyl stearate | 2126 |
| 6 | stearic acid | 2162 |
| 7 | tricosane | 2296 |
| 8 | unknown hydrocarbon | 2695 |
| 9 | unknown hydrocarbon | 2793 |

**Supplementary figure 1:**

Electropalpogram on a pedipalp of an *Araneus diadematus* female in response to sulcatone (left) and the control air (right).

**
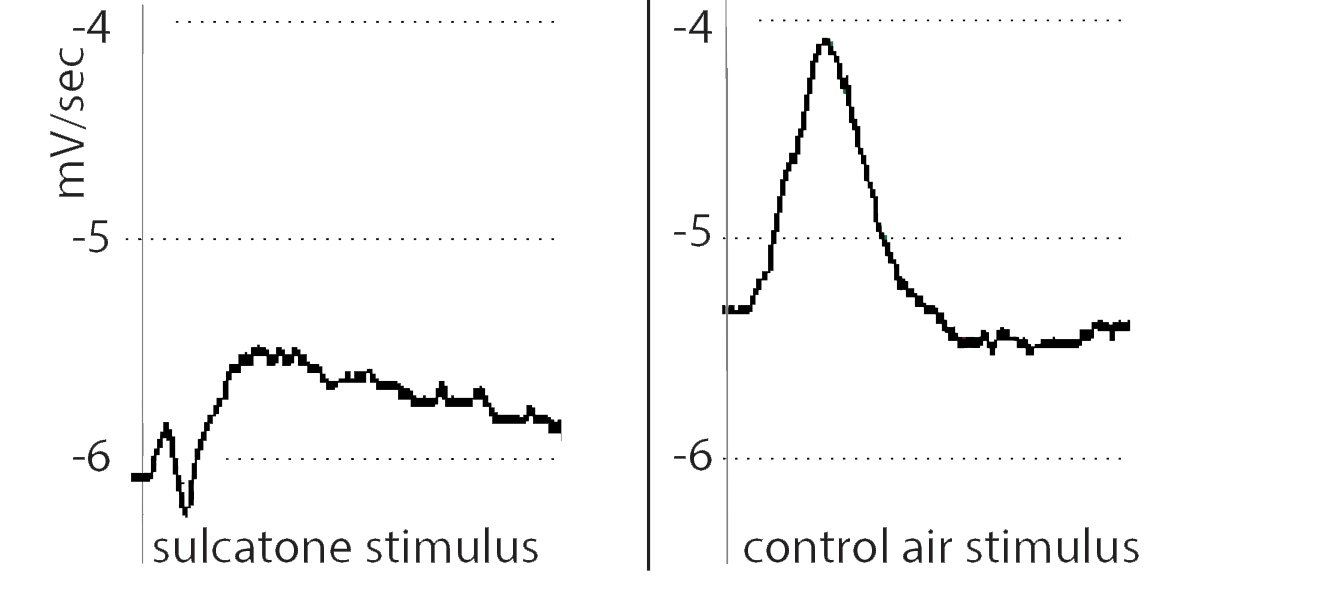
**

**SPSS Output report for the Principal Component Analysis and the Discriminant Function Analysis**

Principal component analysis of the characterized compounds with their loading to the respective component.

| **Compound** | **Loading** | **Component** |
| --- | --- | --- |
| benzyl alcohol | -0.774 | 4 |
| octadecanal | 0.840 | 12 |
| butyl stearate | 0.653 | 1 |
| docosane | 0.775 | 1 |
| tricosane | -0.570 / 0.511 | 11 / 12 |
| butyl octadecenoate | 0.840 | 6 |
| tetracosane | 0.909 | 1 |
| 2-methyltetracosane | 0.920 | 1 |
| pentacosane | 0.813 | 1 |
| hexacosane | 0.505 | 1 |
| 9-, 10-, 11-, 12-, 13-methylhexacosane, mixture | 0.871 | 1 |
| 2-methylhexacosane | 0.894 | 1 |
| 3-methylhexacosane | 0.687 | 1 |
| heptacosane | 0.658 | 1 |
| 11- + 13-methylheptacosane | 0.712 | 8 |
| 2-methylheptacosane | 0.892 | 1 |
| 3-methylheptacosane | -0.573 / 0.523 | 2 / 4 |
| octacosane | 0.722 | 2 |
| 9-, 10-, 11-, 12-, 13-, 14-methyloctacosane | 0.835 | 9 |
| 2-methyloctacosane | 0.750 | 6 |
| dimethyloctacosane and 3-methyloctacosane mixture | -0.773 | 2 |
| nonacosane | 0.517 | 10 |
| 5-methylnonacosane | 0.588 | 8 |
| 2-methylnonacosane and 9,13 dimethylheptacosane, mixture | 0.743 | 1 |
| 3-methylnonacosane | 0.869 | 13 |
| triacontane | -0.651 | 2 |
| 3,13-dimethylnonacosane and 3,X-dimethylnonacosane, mixture | 0.771 | 2 |
| 2-methyltriacontane | 0.728 | 1 |
| 2,X dimethyltriacontane | 0.777 | 7 |
| hentriacontane | -0.609 | 2 |
| unknown alcohol | -0.683 | 2 |
| 9-, 11-, 13-, 15-methylhentriacontane | -0.775 | 5 |
| 13-,17-dimethylhentriacontane and 11-, 15-dimethylhentiacontane and 9-,13-dimethylhentriacontane, mixture | 0.783 | 7 |
| 5,15-, 5,17-dimethylhentriacontane | -0.659 | 2 |
| 3,15-, 3,17-dimethylhentriacontane | 0.690 | 2 |
| 10-, 11-, 12-, 13-, 14-, 15-, 16-methyldotriacontane | 0.583 | 2 |
| 13-,17-dimethyldotriacontane and 11,X-dimethyldotriacontane mixture | -0.892 | 5 |
| hexadecyl hexadecanoate, branched | 0.933 | 10 |
| tritriacontane | -0.592 | 2 |
| unknown alcohol | -0.811 | 2 |
| 11-, 13-, 15-, 17-methyltritriacontane | 0.569 / 0.578 | 2 / 11 |
| 5,15-, 5,17-dimethyltritriacontane | 0.504 / -0.516 | 3 / 4 |
| 3,15-, 3,17-dimethyltritriacontane | 0.643 | 3 |
| 11-, 12-, 13-, 14-, 15-, 16-, 17-methyltetratriacontane | 0.567 / -0.599 | 3 / 4 |
| 13,17-dimethyltetratriacontane and 15,19-dimethyltetratriacontane | 0.793 | 3 |
| 11-, 13-, 15-, 17-methylpentatriacontane | 0.869 | 3 |
| 13,17-, 15,19-dimethylpentatriacontane | 0.909 | 3 |
| unknown hydrocarbon | 0.861 | 3 |
| unknown hydrocarbon | 0.623 / 0.601 | 2 / 3 |
| 13-, 14-, 15-, 16-, 17-, 18-methylhexatriacontane | 0.770 | 6 |
| X,X-dimethylhexatriacontane | 0.897 | 2 |

**Factor Analysis**

| **Total Variance Explained** | | | |
| --- | --- | --- | --- |
| Component | Rotation Sums of Squared Loadings | | |
|  | Total | % of Variance | Cumulative % |
| 1 | 9.961 | 18.112 | 18.112 |
| 2 | 8.582 | 15.603 | 33.715 |
| 3 | 5.914 | 10.752 | 44.467 |
| 4 | 3.780 | 6.872 | 51.339 |
| 5 | 3.204 | 5.825 | 57.164 |
| 6 | 2.705 | 4.919 | 62.083 |
| 7 | 2.571 | 4.675 | 66.758 |
| 8 | 2.031 | 3.692 | 70.450 |
| 9 | 2.005 | 3.646 | 74.096 |
| 10 | 1.844 | 3.353 | 77.449 |
| 11 | 1.818 | 3.306 | 80.755 |
| 12 | 1.673 | 3.041 | 83.796 |
| 13 | 1.629 | 2.963 | 86.758 |

**Discriminant Function Analysis**

| **Tests of Equality of Group Means** | | | | | |
| --- | --- | --- | --- | --- | --- |
|  | Wilks' Lambda | F | df1 | df2 | Sig. |
| REGR factor score 1 for analysis 1 | .972 | .836 | 2 | 57 | .439 |
| REGR factor score 2 for analysis 1 | .710 | 11.665 | 2 | 57 | .000 |
| REGR factor score 3 for analysis 1 | .864 | 4.467 | 2 | 57 | .016 |
| REGR factor score 4 for analysis 1 | .715 | 11.379 | 2 | 57 | .000 |
| REGR factor score 5 for analysis 1 | .907 | 2.907 | 2 | 57 | .063 |
| REGR factor score 6 for analysis 1 | .925 | 2.327 | 2 | 57 | .107 |
| REGR factor score 7 for analysis 1 | .978 | .650 | 2 | 57 | .526 |
| REGR factor score 8 for analysis 1 | .978 | .630 | 2 | 57 | .536 |
| REGR factor score 9 for analysis 1 | .937 | 1.926 | 2 | 57 | .155 |
| REGR factor score 10 for analysis 1 | .945 | 1.667 | 2 | 57 | .198 |
| REGR factor score 11 for analysis 1 | .999 | .023 | 2 | 57 | .977 |
| REGR factor score 12 for analysis 1 | .969 | .918 | 2 | 57 | .405 |
| REGR factor score 13 for analysis 1 | .972 | .831 | 2 | 57 | .441 |

| **Test Results** | | |
| --- | --- | --- |
| Box's M | | 947.751 |
| F | Approx. | 3.393 |
|  | df1 | 182 |
|  | df2 | 7162.962 |
|  | Sig. | .000 |

**Box's Test of Equality of Covariance Matrices**

| **Log Determinants** | | |
| --- | --- | --- |
| Group 1EA, 2ES, 3SA | Rank | Log Determinant |
| adult cuticle extract | 13 | -25.838 |
| subadult cuticle extract | 13 | -1.111 |
| adult silk extract | 13 | -31.110 |
| Pooled within-groups | 13 | -1.488 |

**Summary of Canonical Discriminant Functions**

| **Eigenvalues** | | | | |
| --- | --- | --- | --- | --- |
| Function | Eigenvalue | % of Variance | Cumulative % | Canonical Correlation |
| 1 | 3.480^a^ | 86.4 | 86.4 | .881 |
| 2 | .548^a^ | 13.6 | 100.0 | .595 |
| a. First 2 canonical discriminant functions were used in the analysis. | | | | |
| **Wilks' Lambda** | | | | |
| Test of Function(s) | Wilks' Lambda | Chi-square | df | Sig. |
| 1 through 2 | .144 | 98.752 | 26 | .000 |
| 2 | .646 | 22.277 | 12 | .035 |

| **Standardized Canonical Discriminant Function Coefficients** | | |
| --- | --- | --- |
|  | Function | |
|  | 1 | 2 |
| REGR factor score 1 for analysis 1 | .399 | .008 |
| REGR factor score 2 for analysis 1 | .969 | .435 |
| REGR factor score 3 for analysis 1 | -.430 | .610 |
| REGR factor score 4 for analysis 1 | .846 | -.591 |
| REGR factor score 5 for analysis 1 | -.595 | -.315 |
| REGR factor score 6 for analysis 1 | .566 | .249 |
| REGR factor score 7 for analysis 1 | .334 | -.105 |
| REGR factor score 8 for analysis 1 | .340 | -.071 |
| REGR factor score 9 for analysis 1 | .535 | .207 |
| REGR factor score 10 for analysis 1 | .549 | .001 |
| REGR factor score 11 for analysis 1 | .064 | .021 |
| REGR factor score 12 for analysis 1 | -.222 | .308 |
| REGR factor score 13 for analysis 1 | .353 | .161 |

**Classification Statistics**

| **Classification Function Coefficients** | | | |
| --- | --- | --- | --- |
|  | Group 1EA, 2ES, 3SA | | |
|  | adult cuticle extract | subadult cuticle extract | adult silk extract |
| REGR factor score 1 for analysis 1 | -.292 | .908 | -.847 |
| REGR factor score 2 for analysis 1 | -1.268 | 2.719 | -1.995 |
| REGR factor score 3 for analysis 1 | -.258 | -.846 | 1.517 |
| REGR factor score 4 for analysis 1 | -.082 | 2.035 | -2.685 |
| REGR factor score 5 for analysis 1 | .733 | -1.490 | 1.041 |
| REGR factor score 6 for analysis 1 | -.644 | 1.391 | -1.026 |
| REGR factor score 7 for analysis 1 | -.143 | .723 | -.798 |
| REGR factor score 8 for analysis 1 | -.178 | .747 | -.782 |
| REGR factor score 9 for analysis 1 | -.578 | 1.295 | -.986 |
| REGR factor score 10 for analysis 1 | -.398 | 1.262 | -1.187 |
| REGR factor score 11 for analysis 1 | -.064 | .150 | -.119 |
| REGR factor score 12 for analysis 1 | -.120 | -.414 | .734 |
| REGR factor score 13 for analysis 1 | -.397 | .847 | -.619 |
| (Constant) | -1.669 | -3.630 | -3.969 |

**Classification results**

|  |  | Group 1EA, 2ES, 3SA | Predicted Group Membership | | |
| --- | --- | --- | --- | --- | --- |
|  |  |  | adult cuticle extract | subadult cuticle extract | adult silk extract |
| Original | Count | adult cuticle extract | 18 | 1 | 3 |
|  |  | subadult cuticle extract | 2 | 20 | 0 |
|  |  | adult silk extract | 4 | 0 | 12 |
|  | % | adult cuticle extract | 81.8 | 4.5 | 13.6 |
|  |  | subadult cuticle extract | 9.1 | 90.9 | .0 |
|  |  | adult silk extract | 25.0 | .0 | 75.0 |
| Cross-validated^b^ | Count | adult cuticle extract | 15 | 3 | 4 |
|  |  | subadult cuticle extract | 4 | 18 | 0 |
|  |  | adult silk extract | 7 | 1 | 8 |
|  | % | adult cuticle extract | 68.2 | 13.6 | 18.2 |
|  |  | subadult cuticle extract | 18.2 | 81.8 | .0 |
|  |  | adult silk extract | 43.8 | 6.3 | 50.0 |
